# Does the Medium Matter? Evaluating the Depth of Reflective Writing by Medical Students on Social Media Compared to the Traditional Private Essay Using the REFLECT Rubric

**DOI:** 10.5811/westjem.2019.11.44263

**Published:** 2019-12-19

**Authors:** Alisha Brown, Joshua Jauregui, Jonathan S. Ilgen, Jeff Riddell, Douglas Schaad, Jared Strote, Jamie Shandro

**Affiliations:** * University of Washington, Department of Emergency Medicine, Seattle, Washington; † Keck School of Medicine of University of Southern California, Department of Emergency Medicine, Los Angeles, California; ‡ University of Washington, Department of Biomedical Informatics and Medical Education, Seattle, Washington

## Abstract

**Introduction:**

Social media is a novel medium to host reflective writing (RW) essays, yet its impact on depth of students’ reflection is unknown. Shifting reflection on to social platforms offers opportunities for students to engage with their community, yet may leave them feeling vulnerable and less willing to reflect deeply. Using sociomateriality as a conceptual framework, we aimed to compare the depth of reflection in RW samples submitted by medical students in a traditional private essay format to those posted on a secure social media platform.

**Methods:**

Fourth-year medical students submitted a RW essay as part of their emergency medicine clerkship, either in a private essay format (academic year [AY] 2015) or onto a closed, password-protected social media website (AY 2016). Five raters used the Reflection Evaluation for Learners’ Enhanced Competencies Tool (REFLECT) to score 122 de-identified RW samples (55 private, 67 social media). Average scores on two platforms were compared. Students were also surveyed regarding their comfort with the social media experience.

**Results:**

There were no differences in average composite REFLECT scores between the private essay (14.1, 95% confidence interval [CI], 12.0–16.2) and social media (13.7 95% CI, 11.4–16.0) submission formats (t [1,120] = 0.94, p = 0.35). Of the 73% of students who responded to the survey, 72% reported feeling comfortable sharing their personal reflections with peers, and 84% felt comfortable commenting on peers’ writing.

**Conclusion:**

Students generally felt comfortable using social media for shared reflection. The depth of reflection in RW essays was similar between the private and social media submission formats.

## INTRODUCTION

Reflection provides medical students with opportunities to interrogate their past experiences, puzzle over events that are mentally or emotionally troubling, process the meaning of these experiences, and engage in efforts to transform future actions or attitudes.^1^,^2^ Instilling students with these metacognitive habits has been promoted as a way for them to gain a “greater understanding of both the self and the situation so that future encounters with the situation are informed from previous encounters.”^3^ Following these deliberate metacognitive exercises, students are often prompted to share their personal reflections in the form of reflective writing (RW).^4^–^7^ Ideally, these reflection experiences can help to transform individuals’ attitudes or approaches regarding similar events in the future,^7^ and these new ideas can be expressed to other community members as a means to demonstrate what they have learned.^2^

In medical education, RW narratives are typically shared from students to faculty, then returned by faculty to students with individualized feedback and perspective-sharing.^6^,^8^ While faculty undoubtedly share valuable perspectives with students,^6^,^9^ there are potential opportunities for learning between students that is lost in this curricular structure. Social media provides new ways to think about how reflections can create communities of practice among learners,^5^,^10^–^13^ offering the potential benefits of peer mentorship, broadened perspectives,^14^ and mutual support.^15^–^17^ The conversational nature of social media may allow for more real-time, formative feedback,^11^,^18^ which is likely to be important for deeper reflection.^6^,^8^,^19^ Further, medical students may already be using social media to reflect upon their experiences outside of existing medical school curricula,^20^ potentially highlighting missed opportunities for faculty to explore and enrich students’ perspectives. For example, social media platforms such as FemInEM have provided meaningful spaces for reflection to occur in emergency medicine (EM) outside of the halls of academia.

Moving written reflections from the traditional private paradigm and into a social setting, however, has potential to change how this exercise is experienced by students. Deep reflection requires the learner to engage with his or her sense of self,^7^ and sharing these personal essays with peers may leave students feeling vulnerable to critical peer judgment.^15^ Students may thus choose to censor their deepest thoughts and offer more superficial impressions on social platforms.^10^,^21^,^22^ Further, the framework of sociomateriality would suggest that humans interact with materials (ie, objects, technologies) in critical ways that impact performance and learning.^23^ As such, the mere act of asking students to change the tools they use to document their experiences (eg, private word-processing document vs a social media post) may change how students approach the content, length, and depth of their RW essays.

Given the importance of reflection for medical students’ ongoing professional development^23^,^24^ and the multitude of consequences—both positive and negative—that could result from placing RW essays onto more social platforms, deliberate efforts to understand the effects of these curricular shifts are needed. As such, we were interested in knowing how changing the submission process for RW exercise from a private to a more public format would impact students’ depth of reflections. Using an established scoring rubric,^25^ we sought to compare the depth of reflection in RW essays submitted in a private essay format visible to preceptors only to a new format where RW essays were submitted onto an institutionally-secure social media platform.

**Population Health Research Capsule**

What do we already know about this issue?

*The reflective writing (RW) essay is a common pedagogical tool in medical education. Social media, however, is an increasingly popular venue for physician reflections and may offer a more contemporary setting for students*.

What was the research question?

Does depth of reflection change when students reflect using the traditional essay versus social media?

What was the major finding of the study?

*The depth of reflection was similar between the private and social media submission formats. Students generally felt comfortable using social media for shared reflection*.

How does this improve population health?

*Social media may be a feasible platform to host formal RW exercises, yet issues surrounding mentorship, peer vulnerability, and topic selection should be studied further*.

## METHODS

### Setting

This study took place within the required fourth-year emergency medicine (EM) clerkship at the University of Washington School of Medicine (UW). The majority of UW institution students complete their clinical rotation at two academic urban emergency departments (ED) in Seattle, Washington, although some students elect to rotate in one of 16 community-based clerkship sites across Washington, Wyoming, Alaska, Montana, and Idaho. Clerkship requirements were standardized across training sites. Each student was required to write a RW essay regarding a bioethical dilemma they encountered in the ED during their rotation, and to submit this reflection during week three of their four-week rotation. By way of guidance, students were provided with an RW example written by an emergency physician^26^ and were given the following prompt:^9^

“There are many ethical dilemmas faced in the Emergency Department on a daily basis, such as in this clerkship bioethical reading. Pick one such situation you encountered during your EM clerkship and describe what you learned from it.”

During the 2015 academic year, students submitted RW essays using Microsoft Word (Microsoft Corp., Redmond, WA) format directly to the clerkship directors on a secure, private electronic platform (Catalyst, University of Washington, Seattle, WA). The following academic year (2016), students posted their RWs on a secure social media platform (Yammer, Microsoft Corp., San Francisco, CA), and these samples were visible to their student peers on the rotation, as well as the EM clerkship directors. To facilitate discussion and engagement, students in the 2016 cohort were required to use the online social media platform to respond to at least two of their peers’ RW posts; this assignment was due before the conclusion of their rotation.

We felt that these two student cohorts with comparable preceding clerkship experiences offered an opportunity to examine how students’ depth of reflection might change as a result of this curricular shift. Accordingly, we used the Reflection Evaluation for Learners’ Enhanced Competencies Tool (REFLECT) described by Wald and colleagues^25^ to compare the depth of reflection in RW essays submitted by the 2015 (“private essay”) cohort to those who submitted their essays the following year (“social media” cohort). This study was reviewed by the UW Human Subjects Division and deemed to be exempt based upon its alignment with ongoing curricular evaluation.

### Data Collection

All RW samples from June–September 2015 and June–September 2016 were collected, re-formatted onto a standardized, Word document template and anonymized by a research assistant. All identifying information referencing when or where the student completed the clerkship was removed. The research assistant then assigned a unique, non-consecutive numerical identifier to each RW. Student gender was linked to each de-identified RW in a consolidated database (Excel, OneDrive, Microsoft Corp., Redmond, WA).

### Measures

Word counts were calculated for each RW to gauge differences in essay length between groups. We employed the REFLECT rubric to measure students’ reflective capacity in the RW essays,^25^ an instrument that has existing validity evidence in similar contexts,^27^,^28^ and – in contrast to other tools developed for similar purposes^29^,^30^ – permits greater granularity of assessment across different subdomains of reflection (Appendix 1).^31^ The REFLECT rubric assesses five subdomains of students’ depth of reflection in RW essays: writing spectrum; presence; description of conflict or disorienting dilemma; attending to emotions; and analysis and meaning making. We used working definitions of each of these categories based upon prior descriptions.^25^ Consistent with past use of this tool, raters independently assigned an integer score of 1–4 for each subdomain corresponding to the anchors of “non-reflective,” “thoughtful action or introspection,” “reflection,” and “critical reflection,” respectively. We combined scores for each of the five subdomains to calculate a composite REFLECT rubric score, ranging between 5–20 for each essay.

During the rater training period described below, faculty raters described strong emotional reactions to reading RW pieces. We subsequently decided to record these reactions as a single-item general impression score for each RW essay. General impressions were rated on a three-point scale as negative, neutral, and positive (scored 0–2, respectively).

### Rater Training

Past work with the REFLECT instrument has emphasized the importance of rater training,^31^ with guidance that 4–5 raters, each scoring a minimum of 14 writing samples, were needed to achieve adequate inter-rater reliability (IRR).^27^ In an effort to ensure sufficiently reliable faculty ratings, we trained five faculty raters (AB, JS, JS, JR, JJ), using a sample of RW essays submitted in October 2015 and 2016 (outside of our two RW study periods). Raters independently read the initial published description of the REFLECT rubric^25^ and then scored two representative de-identified RW samples taken from each study cohort. Reviewers met to discuss the rubric and their scoring interpretations; subdomain definitions were subsequently clarified via email communication with the original REFLECT study authors.^32^

To ensure ongoing calibration within our team of raters, a shared document was used across raters to provide clarifications regarding how scores should be applied for each item. Raters then independently coded 10 RW pieces, met to discuss scoring and resolve discrepancies, and again amended the scoring description document. This calibration process was completed twice, for a total of 22 writing samples over three meetings. IRR, as measured by intra-class correlation coefficients (ICC) for the REFLECT rubric scores was calculated sequentially during this training process. Training concluded when the IRR ICC for composite REFLECT score reached 0.80. The ICCs for each subdomain ranged from 0.57–0.86 at the conclusion of rater training (Appendix 2).

### Scoring Period

Following rater training, anonymized RW essays from the 2015 and 2016 enrollment periods were randomly intermixed and sent to reviewers in batches of 25 at timed intervals. Reviewers were blinded to all student characteristics (gender, location of rotation, timing of rotation, essay submission format). Five trained faculty raters independently scored all RW samples and entered scores (REFLECT and general emotional impression) using Google Forms (Alphabet Corp., Mountain View, CA) into an online database. Raters were blinded to each other’s scores, although they met at the approximate halfway point of the study (50 samples) to discuss scoring challenges and improve calibration. To ensure that the reflective writing sample^26^ provided as a prompt to students was illustrative of a “highly reflective” essay, this sample was randomly inserted into the essays scored by three authors unfamiliar with the essay. This essay received an average REFECT score of 19.5 out of 20.

### Post-clerkship Survey

A 12-question electronic survey was developed to gauge students’ perceptions and comfort using the social media platform during the 2016 AY. We developed this survey instrument using guiding principles from Messick’s framework for validity evidence^33^ and survey design best practices.^34^ Survey questions were developed by the study author (AB), drawing from prior work exploring the feasibility of using a social media platform to share reflection,^5^ and then reviewed and revised by five of the authors (AB, JS, JS, JR, JJ). We pilot-tested the survey with four fourth-year medical students (two male, two female) who were not involved in the study, and used a talk-aloud exercise to gather response process validity evidence. Survey questions were revised to ensure clarity. The finalized survey of nine multiple choice and three free-response questions (Appendix 3) was administered in electronic format at the conclusion of the clerkship to all rotating students between June–September 2016. Results were anonymized by the clerkship coordinator, and data were analyzed in aggregate.

### Analyses

Each anonymized RW sample was scored independently by five faculty raters (AB, JS, JS, JR, JJ). REFLECT rubric composite and subdomain scores and general impression scores for each essay were averaged across raters. IRR for the REFLECT composite score, each REFLECT subdomain, and the overall general impression score were calculated using Shrout and Fleiss (2,k) ICCs,^35^ which reflect the reliability of the average score across the five raters (the equivalent of Cronbach’s alpha). We classified these IRR ICCs using criteria proposed by Landis and Koch as fair (ICC values: 0.21– 0.4), moderate (ICC values: 0.41– 0.6), substantial (ICC values 0.61– 0.8)^36^ and include 95% confidence interval (CI). We used descriptive statistics to summarize average composite and subdomain REFLECT scores as well as general impression scores. Average word counts and average REFLECT and general impression scores across the two study periods were compared using two-tailed t-tests with 95% CI; an alpha of 0.05 was considered significant. We correlated average REFLECT composite and general impression scores using Pearson correlation coefficients with 95% CI. We classified correlation coefficients as small (0.10– 0.29), moderate (0.30– 0.49) and large (≥0.50) using thresholds proposed by Cohen.^37^ We used IBM-SPSS Statistics V24 (IBM Corp., Armonk, NY) to perform all analyses.

## RESULTS

A total of 122 RW essays were scored independently by five trained faculty raters: 55 submitted on the private platform and 67 on the social media platform. The demographics of these two student cohorts and average word counts are shown in Table 1. Essay length for the private submission format (480 words, 95% CI, 380–580) was, on average, 14 words longer than those submitted using the social media platform (466 words, 95% CI, 349–582), but this difference was not statistically significant (t [120] = 0.72, p = 0.47). There were no significant differences in word count between genders within the private (p = 0.98) and social media (p = 0.41) submission cohorts.

The five-rater ICC (alpha) for the REFLECT rubric composite scores was substantial (ICC 0.80), as were the ICCs within each of the REFLECT rubric subdomains (ICC range 0.68–0.80). IRR of the general impression was moderate (ICC 0.55). Average overall REFLECT rubric composite scores ranged from 6–20 in the private group and 5–20 in the social media group. There were no significant differences between the composite REFLECT score from the private-submission cohort (14.1, 95% CI 12.0–16.20) and social media (13.7, 95% CI (11.4–16.0) cohorts (t[120] = 0.944, p = 0.35). There were no significant between-group differences in the average scores within each of the five REFLECT rubric subdomains (see Table 2). There were no significant differences in average scores by gender across the entire collection of essays or within each of the two essay submission format cohorts

Average overall rater general impression scores were not significantly different between the private (1.04, 95% CI 0.7–1.4) and social media (0.98, 95% CI 0.6–1.3) submission cohorts (see Table 2). Looked at individually, there was no statistical difference for any subdomain with exception of presence. There was a large degree of correlation between raters’ REFLECT composite and average general impression scores (r = 0.60, p<0.001).

### Survey results

**Figure 1:**
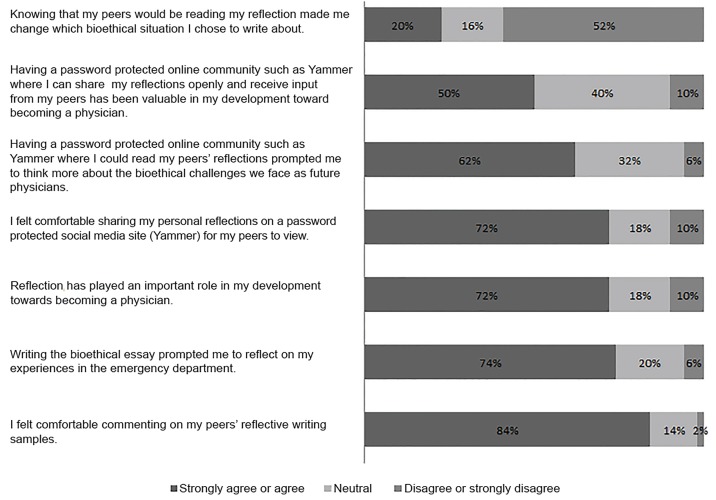
Survey results regarding students’ perceptions and comfort using a social media platform from a study evaluating the depth of reflective writing by medical students on social media compared to the traditional private essay using the REFLECT rubric, 2016. ^a^Reflection Evaluation for Learners’ Enhanced Competencies Tool (REFLECT).

Fifty of 67 students (74.6%) in the social media cohort completed the post-rotation survey (Figure 1). Most students felt comfortable sharing their reflections with peers (72%) and commenting on their peers’ reflections (84%). While 62% of students reported that submitting RW samples on social media prompted them to think more deeply about the bioethical challenges they faced, 20% of students reported changing the content of their essays in response to knowing that peers would be reading their reflections. Ten percent of respondents felt that having a password-protected online community would *not* be a valuable resource for them to share reflections and receive input from peers (50% agreed, 40% neutral). Subgroup analysis revealed no differences in survey results between students who regularly used social media in their personal lives compared to those who did not.

## DISCUSSION

Acts of reflection are conceptualized as deeply personal endeavors.^2^,^7^ Sharing personal reflective writing exercises in social spaces has the potential to foster community and shared learning among peers,^10^,^13^ although the act of sharing risks that students may censor their personal narratives to avoid exposing their deepest feelings with others.^10^,^21^,^22^ Despite these potential influencing factors, our study demonstrated no significant differences in the overall depth of reflection or essay lengths among fourth-year student essays who submitted under private and social formats. Survey results from students who used the social media platform suggest that most felt comfortable sharing their personal reflections and commenting on reflections written by their peers, although it does appear this curricular shift impacted these students in ways that need further exploration.

### Integration with prior work and implications for future research

The lens of sociomaterality enables deliberate consideration of the consequences of introducing a new material or technology,^23^ by exploring how the interplay between the user and the object come together.^38^ This bi-directional interaction between technology and the social person changes interpersonal connections, impacts organizational structures, and shapes the work that these individuals produce.^23^,^38^,^39^ In the context of our curricular shift, we would consider the social media platform as more than an inert technology that passively hosts RW assignments; instead, it becomes an instrument with potential to fundamentally change the practice of reflection itself.

While our study did not show significant differences in the overall depth of reflection as scored by the REFLECT rubric, 20% of our students reported modifying their essay content in consideration of peer viewing. While the majority felt comfortable sharing their essays, it is notable that nearly a third of students felt neutral or uncomfortable with this experience. These findings may suggest that some students would generally prefer to keep their reflections private, although the findings could also align with past research demonstrating the complexity of student peer-to-peer relationships that oscillate between support and judgment.^15^ Did students change their essays because their reflections identified nuances of a case that they had overlooked in the moment? Were there certain topics that inspired new ideas or resonated with individual experiences? Were there certain topics that student felt “safe” or “not-safe” discussing? All of these issues may have influenced the depth and topic choice in complex ways that are not captured in the net neutral effects on the two groups’ average REFLECT scores. A deeper exploration of students’ lived experiences under each of the submission formats would help to elaborate how they balanced these competing tensions.

There are many opportunities to explore how a shift toward making RW exercises more “public” might impact reflection, particularly how faculty input might change students’ experiences of sharing in these new social settings. Faculty feedback is especially critical for learners for whom reflection does not come easily,^3^,^10^,^40^ and effective reflection requires cultivation and mentorship.^41^ Students in our social media group shared and received feedback with peers without a faculty moderator, and the addition of faculty input might have helped these students capitalize on the conversational advantages of this learning platform.^41^,^42^ More regular mentorship on a social media platform may help students recognize the ways in which reflection impacts their personal experiences and the experiences of their peers,^3^ and could help students work toward deeper reflection through formative feedback.^43^ Yet it remains possible that the presence of a faculty moderator in these types of online social forums could also add performance expectations that cause students to withhold particularly sensitive or personally-unflattering disclosures. A richer understanding of how a social media moderator might impact these types of reflective exercises is needed.

By allowing reflections to be visible to peers, many students reported that they continued to engage in reflection about bioethical dilemmas beyond their assigned essay exercise. Although our survey question did not prompt students to distinguish whether their ongoing reflection pertained to the content in their own RW exercise or to RW essays posted by their peers, this finding offers promise. It is quite possible that setting expectations for shared reflection on social media among a community of students and faculty will prompt broader and more regular opportunities for participants to consider and reconsider their challenging professional experiences. The ways that these forms of ongoing shared reflection impact individual members of a community warrant deeper exploration.

## LIMITATIONS

This was a single-center study evaluating a single, reflective-writing sample per student, which limits generalizability. Further, the REFLECT rubric was designed for formative rather than summative evaluation, and thus total scores at a single time point may not accurately reflect students’ growth of reflective capacity.^42^,^43^ While we used mean REFLECT performance metrics as a means to understand performance differences between groups in the context of our curricular shift, this does not fully capture the individualized experiences of our students.

Because our RW was a required clerkship assignment, there may also be a component of performance bias in which students write for approval from their clerkship directors or peers.^44^ That said, the majority of students in the social media group reported that this exercise caused them to reflect, and the assignment was ungraded. For these reasons, we are hopeful that this mitigated these concerns of students “performing” at the expense of true reflection. Finally, it is possible that the rubric itself was not sensitive enough to detect a difference in reflective depth. Two of the rubric domains (ie, “presence” and “analysis and meaning making”) had anchors that were subjective or not well defined, which may explain our lower inter-rater ICC during the study period compared to other studies using the REFLECT rubric.^27^ That said, we used a robust rater-training program and followed pre-existing recommendations for scoring to achieve adequate inter-rater reliability. Our raters’ mean general impressions correlated with mean REFLECT rubric scores, suggesting that these two tools were measuring similar constructs related to faculty members’ impressions of students’ reflections.

## CONCLUSION

Average mean depth of student reflection, as measured by the REFLECT rubric, does not change when students submit reflective-writing essays onto a social media platform compared to submissions sent privately to clerkship directors. While issues of mentorship, peer vulnerability, and topic selection offer opportunities for future exploration, most students feel comfortable sharing reflections and receiving feedback from peers on social media, suggesting this new educational format has potential for future curricula.

## Supplementary Information







## Figures and Tables

**Table 1:** Characteristics of 122 medical student essays submitted in 2015 and 2016 via a traditional, private essay format or social media platform, respectively.

	Private (n=55)	Social media (n=67)	t-value (P value)^a^
Number of essays (% women)	30 (55%)	34 (51%)	
Word count (95% CI)	480 (380–580)	466 (349–582)	t(120)=0.72, p=0.47

Gender Subgroup Analysis			

Word count - women (95% CI)	480 (387–574)	477 (360–595)	
Word count - men (95% CI)	480 (371–588)	454 (337–570)	
t-value (P value)^a^	t(53)=0.03, p=0.98	t(65)= 0.84, p=0.41	

*CI*, confidence interval; *t*, t-value; *p*, p value.

a Significance calculated by comparing private and social media submission using two-tailed t tests with 95% confidence intervals.

**Table 2:** Mean composite and subdomain scores for the REFLECT rubric and average general impression scores for 122 medical student essays submitted in 2015 and 2016 via a traditional, private, essay format or social media platform, respectively.

	Private Cohort Scores (95% CI)	Social Media Cohort Scores (95% CI)	t-value (P value)^b^
Mean REFLECT Composite Scores (IRR 0.80)	14.1 (12.0–16.2)	13.7 (11.4–16.0)	t(1,120)= 0.94, (p=0.35)
Mean REFLECT Subdomain Scores			
Writing spectrum (IRR 0.73)	2.97 (2.5–3.5)	2.95 (2.4–3.5)	t=0.27 (p=0.79)
Presence (IRR 0.80)	3.12 (2.5–3.8)	2.86 (2.3–3.4)	t=2.36 (p=0.02)
Description of disorienting dilemma (IRR 0.68)	2.98 (2.5–3.4)	3.02 (2.5–3.5)	t=−0.44 (p=0.66)
Attention to emotion (IRR 0.79)	2.28 (1.6–2.9)	2.22 (1.5–2.9)	t=0.50 (p=0.62)
Analysis & meaning making (IRR 0.73)	2.73 (2.3–3.1)	2.67 (2.1–3.2)	t=0.75 (p=0.45)
Mean General Impression Scores (IRR 0.55)	1.04 (0.7–1.4)	0.98 (0.6–1.3)	t=1.03 (p=0.31)

*CI*, confidence interval; *t*, t-value; *p*, p value.

The five Reflection Evaluation for Learners’ Enhanced Competencies Tool (REFLECT) subdomains were scored from 1–4 with a maximum composite score of 20, while general Impressions were scored on a three-point scale (0–2).

a The inter-rater reliability (IRR) for five faculty raters was calculated using intraclass correlation coefficients.

b Significance was calculated by comparing private and social media scores using two-tailed t tests.
