# Supplementary material for: Does the Medium Matter? Evaluating the Depth of Reflective Writing by Medical Students on Social Media Compared to the Traditional Private Essay Using the REFLECT Rubric
Source: West J Emerg Med. 2019 Dec 19;21(1):18–25. doi: 10.5811/westjem.2019.11.44263 (PMC6948677; doi:10.5811/westjem.2019.11.44263)
Supplement: Supplementary file 1 [file wjem-21-18-s001.docx]

**Appendix 1:** The Reflection Evaluation for Learners’ Enhanced Competencies Tool (REFLECT).

|  | **Level** | | | | | **Axis II for critical reflection** | |
| --- | --- | --- | --- | --- | --- | --- | --- |
| ***Criterion*** | | ***Habitual action***  ***(Nonreflective)*** | ***Thoughtful action***  ***or introspection*** | ***Reflection*** | ***Critical reflection*** | ***Transformative***  ***reflection and***  ***learning*** | ***Confirmatory***  ***learning*** |
| **Writing spectrum** | | Superficial descriptive  writing approach (fact reporting, vague impressions) without reflection or introspection | Elaborated descriptive  writing approach and impressions without reflection | Movement beyond reporting or descriptive writing to reflecting (i.e., attempting to understand, question | Exploration and critique of assumptions, values, beliefs, and/or biases,  and the consequences of action (present and future) | Frames of reference or  meaning structures are  transformed. Requires  critical reflection Integration of new learning into one’s identity, informing future perceptions, emotions, attitudes, insights, meanings, and actions. Conveys a clear sense of a breakthrough | Frames of reference or  meaning structures are  confirmed. Requires  critical reflection |
| **Presence** | | Sense of writer being partially present | Sense of writer being partially present | Sense of writer being largely or fully present | Sense of writer being fully present |  |  |
| **Description of conflict**  **or disorienting**  **dilemma** | | No description of the disorienting dilemma, conflict, challenge, or  issue of concern | Absent or weak description of the disorienting dilemma, conflict, challenge, or issue of concern | Description of the  Disorienting dilemma, conflict, challenge, or issue of concern | Full description of the disorienting dilemma, conflict, challenge, or issue of concern that includes multiple perspectives, exploring alternative explanations, and challenging assumptions |  |  |
| **Attending to emotions** | | Little or no recognition or attention to emotions | Recognition but no exploration or attention to emotions | Recognition, exploration, and attention to emotions | Recognition, exploration, attention to emotions, and gain of emotional insight |  |  |
| **Analysis and meaning**  **making** | | No analysis or meaning making | Little or unclear analysis or meaning making | Some analysis and meaning making | Comprehensive analysis and meaning making |  |  |
| **Optional minor**  **criterion: Attention to**  **assignment (when**  **relevant)** | | Poorly addresses the assignment question and does not provide a compelling rationale for choosing an alternative | Partial or unclear addressing of assignment question; does not provide a compelling rationale for choosing an alternative | Clearly answers the  assignment question or, if  relevant, provides a compelling rationale for choosing an alternative | Clearly answers the  assignment question or,  if relevant provides a  compelling rationale for  choosing an alternative |  |  |

Adapted from: Wald HS, Borkan JM, Taylor JS, Anthony D, Reis SP. Fostering and evaluating reflective capacity in medical education: developing the REFLECT rubric for assessing reflective writing. Acad Med. 2012;87:41-50.
