# Supplementary material for: Does the Medium Matter? Evaluating the Depth of Reflective Writing by Medical Students on Social Media Compared to the Traditional Private Essay Using the REFLECT Rubric
Source: West J Emerg Med. 2019 Dec 19;21(1):18–25. doi: 10.5811/westjem.2019.11.44263 (PMC6948677; doi:10.5811/westjem.2019.11.44263)
Supplement: Supplementary file 2 [file wjem-21-18-s002.docx]

**Appendix 2.** Inter-rater reliability of REFLECT^a^ rubric during the training period for five faculty raters.

|  | **Inter-rater reliability^b^** |
| --- | --- |
| **REFLECT Composite Score** | 0.80 |
| **REFLECT Subdomain Scores** |  |
| ***Writing spectrum*** | 0.61 |
| ***Presence*** | 0.86 |
| ***Description of disorienting dilemma*** | 0.68 |
| ***Attending to emotion*** | 0.84 |
| ***Analysis and meaning making*** | 0.57 |

Notes: ^a^Reflection Evaluation for Learners' Enhanced Competencies Tool (REFLECT). ^b^ Inter-rater reliability was calculated using intraclass correlation coefficients.
