# Supplementary material for: Does the Medium Matter? Evaluating the Depth of Reflective Writing by Medical Students on Social Media Compared to the Traditional Private Essay Using the REFLECT Rubric
Source: West J Emerg Med. 2019 Dec 19;21(1):18–25. doi: 10.5811/westjem.2019.11.44263 (PMC6948677; doi:10.5811/westjem.2019.11.44263)
Supplement: Supplementary file 3 [file wjem-21-18-s003.docx]

**Appendix 3.** Survey Questions for a medical student cohort who used a social media platform (Yammer) as a means to a host reflective writing assignments during their emergency medicine clerkship in 2016.

**The following pertains to your development as a physician**

1. Reflection has played an important role in my development towards becoming a physician
   1. Strongly Agree
   2. Agree
   3. Neutral
   4. Disagree
   5. Strongly Disagree
   6. Comments _____________________________

**The following questions pertain to your bioethical reflective writing piece.**

1. Writing the bioethical essay prompted me to reflect on my experiences in the ED.
   1. Strongly Agree
   2. Agree
   3. Neutral
   4. Disagree
   5. Strongly Disagree
   6. Comments _____________________________
2. Having a password protected community such as Yammer where I can share my reflections openly and receive input from my peers has been valuable in my development toward becoming a physician.
   1. Strongly Agree
   2. Agree
   3. Neutral
   4. Disagree
   5. Strongly Disagree
   6. Comments _____________________________
3. Having a password protected online community where I could read my peers’ reflections prompted me to think more about the bioethical challenges we face as future physicians.
   1. Strongly Agree
   2. Agree
   3. Neutral
   4. Disagree
   5. Strongly Disagree
   6. Comments _____________________________
4. I felt comfortable sharing my personal reflections on a password protected social media site (Yammer) for my peers to view.
   1. Strongly Agree
   2. Agree
   3. Neutral
   4. Disagree
   5. Strongly Disagree
   6. Comments _____________________________
5. I felt comfortable commenting on my peers’ reflective writing samples.
   1. Strongly Agree
   2. Agree
   3. Neutral
   4. Disagree
   5. Strongly Disagree
   6. Comments _____________________________
6. Knowing that my peers would be reading my reflection made me change which bioethical situation I chose to write about.
   1. Strongly Agree
   2. Agree
   3. Neutral
   4. Disagree
   5. Strongly Disagree
   6. _____________________________
7. If you answered Strongly Agree or Agree in question 7, why did it change and how did it change?
8. What do you see as the benefits of using a secure social media platform like Yammer for reflective writing on your clinical experiences?
9. What do you see as the drawbacks of using a secure social media platform like Yammer for reflective writing on your clinical experiences?

**The following set of questions pertain to your experiences outside of class assignments and work.**

1. Outside of assignment purposes or work purposes, I use social media (such as Facebook, Instagram, Twitter, etc) to reflect on my life experiences.
   1. I use it regularly to reflect publicly
   2. I use it rarely to reflect publicly
   3. I never use it to reflect publicly
2. How I interact with my professional peers (i.e. medical students, residents etc) online for school and work is the same as how I interact with them online outside of school and work.
   1. Strongly Agree
   2. Agree
   3. Neutral
   4. Disagree
   5. Strongly Disagree

_____________________________
